# Nitric Oxide in Seed Biology

**DOI:** 10.3390/ijms232314951

**Published:** 2022-11-29

**Authors:** Katarzyna Ciacka, Pawel Staszek, Katarzyna Sobczynska, Urszula Krasuska, Agnieszka Gniazdowska

**Affiliations:** Department of Plant Physiology, Institute of Biology, Warsaw University of Life Sciences, Nowoursynowska 159, 02-776 Warsaw, Poland

**Keywords:** aging, dormancy, germination, nitric oxide, phytohormones, priming, ROS, seed vigour

## Abstract

Nitric oxide (NO) has been recognized as a gasotransmitter in the mainstream of plant research since the beginning of the 21st century. It is produced in plant tissue and the environment. It influences plant physiology during every ontogenetic stage from seed germination to plant senescence. In this review, we demonstrate the increased interest in NO as a regulatory molecule in combination with other signalling molecules and phytohormones in the information network of plant cells. This work is a summary of the current knowledge on NO action in seeds, starting from seed pretreatment techniques applied to increase seed quality. We describe mode of action of NO in the regulation of seed dormancy, germination, and aging. During each stage of seed physiology, NO appears to act as a key agent with a predominantly beneficial effect.

## 1. Introduction

### 1.1. Historical View of NO Plant-Related Literature

Many papers have emphasized the role of various gasotransmitters in plant ontogenesis and reaction to environmental stresses [1,2]. A prominent group of these compounds is reactive nitrogen species (RNS), the most frequently studied of which is nitric oxide (NO).

Since the late 1950s, more than 15,000 scientific articles related to RNS action or detection in various plant materials are listed in the Web of Science database. Most of this research concerned the use of plant tissue extracts (including seed extracts) in the analysis of NO metabolism in animal models. Among these publications, 37% are related to plant science, 18% to biochemistry and molecular biology, and 11% to pharmacology and pharmacy (according to Web of Science Categories, Table S1).

**Figure 1:**
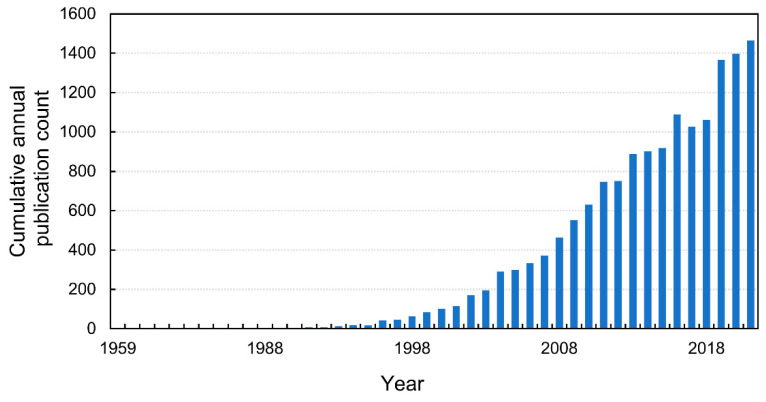
The cumulative annual publication count of papers on NO in plant science. Details of the search are available in the Supplementary Materials.

In plant science, the first mention of the use of NO in the literature appeared in 1959 (Figure 1) in a paper describing the influence of exogenous NO on creeping bentgrass (*Agrostis stolonifera* L.) seeds in response to radiation [3]. However, since its publication, there has been no particular increase in interest in the role of RNS in the regulation of plant growth and development (Figure 1). The first breakthrough research on NO formation in plant cells was published by Klepper [4] and referred to the release of NO from herbicide-treated soybean (*Glycine max* (L.) Merr.) leaves. Since the 1990s, there has been a gradual increase in the number of publications on metabolism and the signalling role of NO in plant biology (Figure 1). Then, an inventive paper by Delledonne et al. was published in *Nature* [5] about the role of NO in plant disease resistance, which has a current citation index of ca. 1350 (according to a search performed on 13 October 2022). In the same year, the Nobel Prize in Physiology or Medicine was awarded for research on NO as a signalling molecule in the cardiovascular system [6].

**Figure 2:**
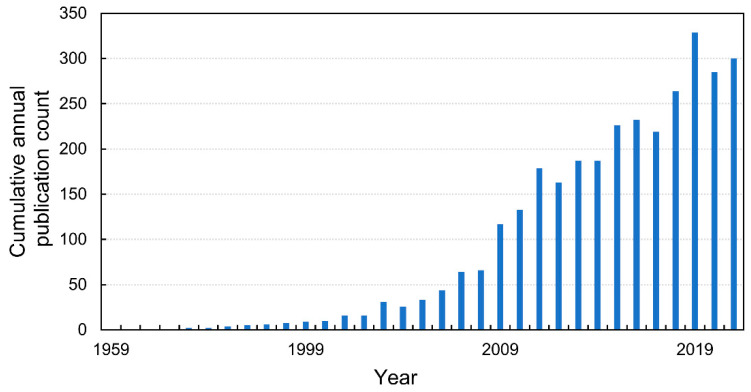
The cumulative annual publication count of papers on NO and seeds. Details of the search are described in the Supplementary Materials.

Between 1900 and 2021, an analysis of studies reporting on NO and seeds in the Web of Science database showed a total of 3300 records (Figure 2), with the first record in 1959. Most records are related to plant sciences (29%), biochemistry and molecular biology (17%), pharmacology and pharmacy (11%), and food science technology (9%). According to the query used (details in Table S2), the analysis showed that the first article on the impact of NO on seed germination was published by Giba et al. [7]. However previously, despite the use of NO donors, the role of NO in the regulation of seed biology had not been underlined. Therefore, the applied query ((ALL = (“seed” OR “seeds”)) AND ALL = (“reactive + nitrogen + species” OR “RNS” OR “nitrosative + stress” OR “nitric + oxide”)) did not return such studies. An example is a paper published in 1990 that concerned the germination of princess tree (*Paulownia tomentosa* (Steud.)) seeds. The authors demonstrated that after using 10 mM KNO_3_ (as a NO donor), a single red-light pulse was sufficient to induce seed germination [8]. Not only the direct treatment of seeds with NO donors but also the fertilization of the mother plant with nitrogen influences the germination of the seeds [9]. On the other hand, atmospheric NO of various origins seems to be important for the regulation of seed biology [10].

### 1.2. NO Sources in Seeds and NO Donors Used in Laboratory Practice

A molecule of NO consists of nitrogen and oxygen atoms. Owing to the presence of unpaired electrons in NO radical form, it exerts a high reactivity. The biochemistry of NO is also linked to the formation of nitrosonium cations (NO^+^) and nitroxyl anions(NO^−^). NO reacts with molecules that also possess unpaired orbital electrons or transition metal ions. When it is converted to other RNS, it may react with nucleic acid, proteins, and lipids. Easy diffusion through membranes supports its reactivity [11].

Since early research on the role of NO in plants, the biosynthesis of this gaseous molecule has been at the centre of attention. NO is produced in plants via many enzymatic and nonenzymatic pathways, the functioning of which depends on plant tissue, developmental stage, and environmental factors. The list of NO biosynthesis pathways is not exhaustive, and the activity of some of them remains the topic of active discussion [12,13,14,15].

Although the pathways of NO biosynthesis in seeds have been the subject of investigation for years, many questions remain to be answered. Understanding this phenomenon thoroughly is challenging due to the alternation in oxygen levels in the tissue during seed germination (imbibition). The first information on this subject was published in 2004. Then, the non-enzymatic reduction of nitrite (NO_2_^−^) to NO was demonstrated in the apoplast of the aleurone layers of germinating barley (*Hordeum vulgare* L.) caryopsis [16]. Moreover, nitrate reductase (NR)-dependent NO formation was confirmed in sorghum (*Sorghum bicolor* L. Moench) seeds [17]. Other NO producers in seeds include mitochondria [18]. NO generation in the reaction catalysed by NR or in mitochondria requires a low oxygen concentration, representing a potential source of this compound in the early stages of seed germination when oxygen depletion occurs [19]. As a result of limited oxygen access, the oxidative pathways (via nitric oxide synthase-like activity or hydroxylamine oxidation) of NO biosynthesis are unlikely to be active at the onset of germination. However, after loosening the seed coat or during radical protrusion, these routes could be predominant [20].

For years, the question has been raised as to the importance of endogenous NO in the regulation of seed dormancy and germination, as well as the importance of the operation of external sources of this gas transmission. Gupta et al. [21] presented a list of the most commonly used NO donors, which is reproduced in Table 1. Some NO donors such as sodium nitroprusside (SNP), one of the cheapest NO sources, are very controversial [22].

## 2. Seed Priming or Presoaking with SNP Used as NO Donor Increases Seed Germination and Seedling Tolerance to Various Stresses

Seed priming techniques are used to increase seed vigour and germination and decrease seedlings’ susceptibility to various environmental stresses. Thus, diverse techniques of seed priming are commonly used by seed companies worldwide as promising methods to increase crop productivity under inconvenient environmental conditions. Priming seems to act as a treatment that activates a kind of “cellular memory” that allows developing seedlings to become more tolerant to stressors [34]. Generally, seed priming is defined as “pre-germination hydration” that induces various physiological, biochemical, and molecular events leading to the accomplishment of germination under favourable moisture conditions and the formation of properly formed, healthy seedlings. This technique is based on controlled seed hydration sufficient to permit pregerminative metabolic events to proceed but insufficient to allow for radicle protrusion. After priming, seeds are dried back to their initial water content and can be stored and/or sown via conventional techniques [35]. As the impact of NO on the increased tolerance of seedlings or plants to stresses has been proven by many authors [36,37,38], some NO donors (predominantly SNP) were tested using priming techniques. NO donors are used separately or in combination with other molecules belonging to, e.g., reactive oxygen species (ROS). Habib et al. [39] used 100 µM SNP separately or in combination with hydrogen peroxide (H_2_O_2_) to test the beneficial effect of these two molecules on wheat (*Triticum aestivum* L.) under water deficit conditions. Combined H_2_O_2_ and SNP soaking of rapeseed (*Brassica napus* L.) seeds resulted in effective protection against salt (NaCl) stress [40]. A presowing exposure of seeds to a magnetic field resulted in enhanced germination and emergence [41], and Patel et al. [42] demonstrated that SNP (100–500 µM) enhanced germination and vigour of magnetoprimed maize (*Zea mays* L.) seeds.

**Figure 3:**
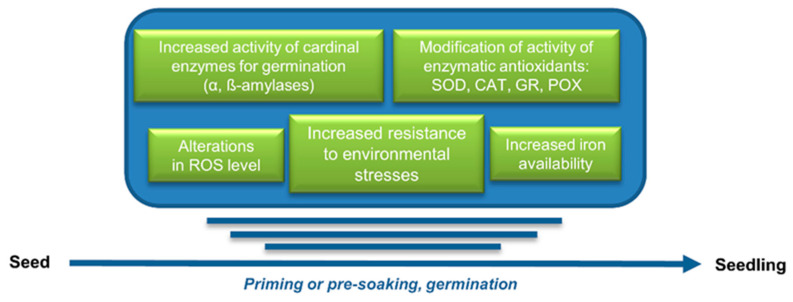
NO (mostly SNP)-induced changes observed after seed priming or seed presoaking resulting in stimulation of seed germination and enhancement of seedling growth under unfavourable environmental conditions. **The order of incidents shown in the figure does not reflect the actual sequence of events**.

Many researchers have tested SNP using seed presoaking techniques to increase the seed germinability, vigour, and resistance of developing seedlings to stressors, particularly unfavourable environmental conditions. SNP (10–100 µM) was used as a priming factor to improve wheat seedling tolerance to chilling stress [43]. Soaking of seeds of two rapeseed cultivars (differing in susceptibility toward stresses) in SNP increased seed germination parameters under low-temperature and drought conditions [44]. Carrot (*Daucus carota* L.) seeds subjected to water deficit germinated better after the application of 100–300 µM SNP [45]. Similarly, soaking of *Cynanchum bungei* Decne seeds in SNP accelerated seed germination and increased germination and vigour under salinity [46]. Priming of lettuce (*Lactuca sativa* L.) seeds in SNP had a beneficial effect on copper toxicity, although no effect of SNP was observed under non-stressed conditions [47]. In Indian mustard (*Brassica junea* L.) seeds, pretreatment with 100 µM SNP increased germinability in excess copper [48]. The protective action of SNP was also demonstrated in sesame (*Sesamum indicum* L.) seeds exposed to cadmium [49]. Wheat seeds of seven different genotypes primed with 10 or 100 µM SNP germinated well at low temperatures [43]. An induction of chilling tolerance was observed in seeds of another wheat genotype and young seedlings after presoaking of caryopsis with 100 µM SNP [50]. Increased low-temperature tolerance was also reported for tomato *(Solanum lycopersicum* L.) seeds after priming with 200 µM SNP [51]. Priming of Brachiaria grass (*Urochloa brizantha* (Hochst. ex A. Rich.) R. Webster) seeds with SNP (100 µM) induced protective action against water restriction and salinity [52]. In some reports, authors confirmed the positive action of NO after using SNP solution by applying a NO scavenger (2-(4-carboxyphenyl)-4,4,5,5-tetramethylimidazoline-1-oxyl-3 oxide; cPTIO), an inhibitor of NO synthase (L-N^G^-nitro arginine methyl ester), an inhibitor of NR (sodium tungstate), or methylene blue, which is regarded as a blocker of NO action [42,51,53]. Most of these reports share a common mechanism of NO action: increased α-amylase and ß-amylase activity, enhanced starch degradation leading to increased carbohydrate concentration, and activation of the enzymatic cellular antioxidant system [42,44,49,51,54]. A summary of experimentally confirmed events occurring in seeds after priming or presoaking in the presence of SNP used as a NO donor is shown in Figure 3.

Some examples of the potential application and perspectives of the utilization of various NO donors in agriculture were recently reviewed [55,56,57]. Tests of SNP presoaking of seeds of common crops, such as sugar beet (*Beta vulgaris* L.), Chinese cabbage (*Brassica rapa* L. subsp. *pekinensis*), and barley (*Hordeum vulgare* L.), pointed to the effectiveness of this chemical for seed quality improvement [58].

All data on the application of SNP as a priming agent or presoaking solution have to be analysed in the context of the current knowledge on the use of SNP as a NO donor. It is debatable whether SNP can be considered a NO donor only [22,59,60,61]. SNP releases a substantial amount of cyanide and iron; thus, its use in NO research as a NO donor is doubtful [61]. Furthermore, in most cited papers referring to seed priming or presoaking with SNP, no detailed information was included on the light conditions of SNP treatment. Taking into account that SNP in aqueous solution was degraded when exposed to white or blue light but not to red light or in darkness [62], the effect of SNP but not strictly NO should be considered, and the results of cited reports should be interpreted with caution. However, the possibility of the use of SNP as a NO donor for seed quality improvement by priming and presoaking is still promising. Recently, gas priming with acidified KNO_2_ was proposed as a novel treatment for air-dried seeds in research on dormancy release and seed germination [63]. As these chemicals (SNP and KNO_2_) are relatively inexpensive, they have a putative potential for such applications. Such treated seeds should not be stored for a long time but be quickly distributed for seedlings production. Detailed studies on a large scale for a larger batch of seeds should be performed before commercialisation.

## 3. NO-Phytohormone Network in Seed Dormancy and Germination

Seed germination includes events preceding cell growth and differentiation, starting from seed imbibition and leading to the induction of elongation growth of the embryonic axis [64]. The beginning of growth, manifested by the breakage of the seed coat by the embryonic axis, indicates the end of germination and the beginning of seedling growth. However, not all viable seeds separated from the mother plant germinate successfully, owing to seed dormancy, which is the physiological state resulting from the genotypic properties of the seeds and/or environmental factors disrupting germination [64].

Various NO donors have been shown to reverse dormancy and stimulate seed germination in many plant species [65,66]. Most seeds, whose dormancy is reversed by the use of NO donors exhibit physiological dormancy associated with an imbalance between stimulators and inhibitors of germination. This type of dormancy is alleviated by NO in apple (*Malus domestica* Borkh.) embryos, redroot pigweed (*Amaranthus retroflexus* L.), Arabidopsis (*Arabidopsis thaliana* L.) Heynh.), barley, empress tree (*Paulownia elongata* S.Y.Hu), and switchgrass (*Panicum virgatum* L.) seeds [53,67,68,69,70,71]. NO-dependent removal of secondary dormancy (resulting from unfavourable environmental conditions) of wild cabbage *(Brassica oleracea* L.) was also reported [72]. The influence of various donors of NO on the thermodormancy and photodormancy of lettuce (cv. ‘Jianye Xianfeng No. 1′) seeds was reported by Deng and Song [73]. Bethke et al. [67,68,74] used an experimental model in which the seeds of Arabidopsis germinated in the presence of vapours released from an aqueous solution of SNP. They observed that cPTIO strengthened the dormancy of Arabidopsis seeds but did not impact the dormancy of non-dormant seeds [67]. The positive impact of SNP on germination was reported for chickweed (*Stellaria media* (L.) Vill) seeds [75]. SNP (10 to 400 µM) also increased the germination of flixweed (*Descurainia sophia* (L.) Webb ex Prantl) seeds [76]. The use of an SNP solution (0.1–800 µM) resulted in a stimulating effect on the germination of yellow lupine (*Lupinus luteus* L.) seeds and decreased their sensitivity to heavy metals (lead and cadmium), as well as salinity [77]. Similarly, SNP (50 and 200 µM) stimulated the germination of *Suaeda salsa* (L.) Pall. seeds under salinity [78] and the germination of tomato seeds under osmotic stress [79]. Seed germination of grasses such as millet (*Panicum virgatum* L.), big bluestem grass (*Andropogon gerardii* Vitman), and yellow Indian grass (*Sorgastrum nutans* L. Nash) was stimulated by 200 µM SNP [69]. This effect was reversed by 200 µM PTIO. Furthermore, NO was reported to be released as a result of the acidification of NO_2_^−^, with a much better effect than SNP on the seed germination of various plant species [69].

The mechanism of NO action in seed dormancy removal is connected to the regulation of phytohormone content and signalling (Table 2, Table 3, Table 4 and Table 5). Abscisic acid (ABA) is responsible for dormancy induction and maintenance [66]. A decrease in ABA content was detected after NO application in freshly harvested Arabidopsis seeds or dormant wild cabbage seeds [72,80]. The triple-mutation *nia1nia2noa1* manifested by a deficiency in NO generation was related to delayed germination, as well as seed hypersensitivity to ABA during the germination and early seedling development stages [81]. Moreover, the treatment of dormant apple embryos with SNP under light conditions resulted in a decrease in embryo sensitivity to exogenous ABA [70,82]. For dormant tissue (apple embryos or non-after-ripened hedge mustard (*Sisymbrium officinale* (L.) Scop.) seeds), NO impacted ABA levels via downregulation of *NCED* gene expression (related to ABA biosynthesis) [83,84]. Generally, NO-dependent stimulation of seed germination of dormant or nondormant seeds/embryos is associated with an upregulation of *CYP7072A* transcript levels (related to ABA degradation) (Table 2). Research performed on Arabidopsis seeds revealed that an increase in *CYP707A2* expression by NO_3_^-^ requires the presence of NIN-like protein 8, which directly binds to the promoter of the gene and ultimately reduces the ABA level [85]. Moreover, NO initiates post-translational modifications (PTMs) of proteins of the ABA biosynthesis and signal transduction pathways (via *S*-nitrosation or nitration), disturbing the accumulation and mode of action of this phytohormone, reviewed in detail in [86]. ABA signalling is also downregulated by NO-mediated degradation of VII ethylene response transcription factor (ERFVII), a positive regulator of ABI5 (the element of ABA signal transduction), via the N-end rule pathway of proteolysis [86,87].

A genome-wide association study of barley genotypes revealed that NO release (highly abundant in non-dormant genotypes) was associated with *GA2ox4*, *GA20ox3*, and *GA20ox1*, which played a key role in gibberellin (GA) biosynthesis [90]. This phytohormone regulates the loss of seed dormancy and promotes seed germination [86]. The conversion of inactive forms of GAs to active forms requires the presence of GA 3-oxidase (GA3ox). On the other hand, GA 2-oxidase (GA2ox) is responsible for GA deactivation. In hedge mustard seeds [83,88], NO promoted the germination of after-ripened and non-after-ripened seeds through transcriptional changes of various genes of GA metabolism. The simultaneous application of KCN (dormancy-removing agent) and cPTIO decreased the *GA3ox* levels in Arabidopsis seeds (compared to the effect obtained with HCN only) (Table 3), confirming the role of NO in the regulation of gene expression of GA biosynthesis [91]. NO may also influence GA signalling through a decrease in the *RGL2* transcript level, as detected for hedge mustard seeds, irrespective of the depth of dormancy [83,88]. As this gene encodes a protein belonging to the DELLA family [86], a repressor of the GA transduction pathway, NO may promote the expression of genes induced by GA.

The mechanism of ethylene (ET) action and its cooperation with NO during dormancy alleviation and seed germination is less elaborated. Pretreatment with NO (acidified KNO_2_) followed by ET application increased the germination of redroot pigweed seed by 30% [71]. In the same research, a stimulatory effect of NO and aminocyclopropane-1-carboxylic acid (ACC, the precursor of ET) on germination was associated with increased ET levels before radicle protrusion. Similarly, when the culture of NO-treated apple embryos was prolonged, a gradual increase in ET emission was detected [82]. The stimulation of ET production in wild cabbage seeds after NO treatment was related to an increase in the expression of genes encoding proteins involved in its biosynthesis (ACC synthase and oxidase) and signalling (ethylene receptor) (Table 4) [72]. The positive effect of NO application on seed dormancy removal of wild cabbage was disrupted after the use of 2,5-norbornadiene, an ET binding inhibitor. The link between NO, ET, and polyamines (PAs) in the regulation of apple embryo germination was mentioned by Krasuska et al. [92].

Various effects of jasmonates (JA) on the germination of seeds of many species were detected in [93]. JA stimulated the germination of dormant apple embryos [94] but inhibited the germination of non-dormant embryos. A fluctuation in JA content was observed in apple embryos during dormancy removal by cold stratification [95]. Only a few reports have indicated the interaction of JA and NO in seed biology. In axes of apple embryos, NO treatment increased the transcript level of *AOS* (related to JA biosynthesis) and decreased the transcript level of *JAZ3* (a negative regulator of JA signalling) (Table 5), suggesting the stimulation of JA production and the induction of the transduction pathway of this phytohormone [84]. Similarly, the results of the expression of genes related to the formation of JA derivatives indicate that the NO-induced transition of apple embryos from a dormant to non-dormant state was accompanied by the formation of methyl jasmonate (MeJa; upregulation of *JMT*) rather than JA-isoleucine synthesis (downregulation of *JAR1*) (Table 5). Jacobsen et al. [93] confirmed the importance of NO–JA interactions in seeds, reporting that MeJa reversed the inhibitory effect of blue light on the germination of wheat grains, and its action was NO-related.

## 4. NO–ROS Link in the Regulation of Seed Biology

In seeds, the most frequently studied ROS are hydroxyl radicals (^•^OH), superoxide anions (O_2_^•−^), or H_2_O_2_, and their concentration is controlled by an antioxidant system [96]. Catalases (CAT), glutathione reductases (GR) or superoxide dismutases (SOD) are included in the group of enzymatic antioxidant, whereas non-enzymatic antioxidants include ascorbic acid (AsA) or glutathione (GSH) in reduced form and tocopherols [57,97]. In seed physiology, a dual role of ROS (depending on the actual concentration) is described by the model of the oxidative window. The phrase “oxidative window” is used in reference to germination-optimal ROS concentration. An ROS concentration below or above the “oxidative window” impairs the seed’s ability to germinate, as well as seed vigour, and disturbs the development of additional seedlings [97,98]. RNS are produced in cellular compartments, which are typically regarded as sources of ROS [99]. Concomitantly formed ROS and RNS interact with each other; thus their concentration, is interdependent [100]. The indirect regulation of ROS concentration by NO may be related to the NO-dependent control of enzymes involved in ROS metabolism. The impact of NO on the activity of basic enzymatic antioxidants SOD, CAT, glutathione peroxidase-like, and GR was noticed observed the dormancy release of apple embryos [101]. The regulation of the activity of antioxidant enzymes may be a manifestation of NO-mediated PTMs, as confirmed in other plant tissues [102].

The physiological (ontological) fate of seeds is determined by both ROS and RNS [103]. Similar action of the reactive compounds was demonstrated for seed dormancy loss of many plant species, including apple, sorghum, or barley [17,101,104,105]. The first important data on seed germination related to the increase in NO synthesis and its effect on ROS concerned sorghum seeds [17], revealing that ROS in germinated seeds operate with NO. During germination sensu stricto of sorghum seeds (24 h), high NO liberation was correlated with a non-detectable level of lipid radicals. In contrast, in the postgermination phase (48 h) a decline in cellular NO level was accompanied by an increased amount of lipid radicals in embryonic axes [17]. Further results showed that sorghum seed treatment with NO donors (1 mM SNP or 1 mM DETA-NONOate) kept RNS at a relatively high concentration (a “steady-state” level), stimulating seed germination [106]. This beneficial role of NO was linked to protection against oxidative damage by a decline in the level of carbonylated proteins and the maintenance of membrane integrity by reducing the formation of lipid radicals [106]. Moreover, a NO-positive action was associated with increased iron availability for the labile iron pool, which is necessary for the promotion of cell development, accompanied by reduced iron toxicity (linked to ^•^OH formation during the Fenton reaction). NO–ROS cooperation during germination was also proven for switchgrass and Arabidopsis seeds [107,108]. In these seeds, PTIO diminished the stimulatory effect of H_2_O_2_ on seed germination. Moreover, for Arabidopsis seeds, a significant increase in the expression of *CYP707A2* was obtained after SNP or H_2_O_2_ treatment [108]. However, with the simultaneous application of H_2_O_2_ and PTIO, this effect was abolished, as reflected by ABA accumulation. Therefore, the authors proposed that H_2_O_2_-dependent ABA catabolism during the regulation of dormancy and germination of Arabidopsis seeds requires NO [108].

Dormancy alleviation in apple and barley embryos was associated with alterations in NO and ROS levels [105,109]. Short-term treatment of apple embryos with different dormancy-reversing factors (e.g., HCN, NO donors, ET donors, and PA) increased not only ROS content but also NO emission [82,101,104,110]. Moreover, the results presented by Krasuska et al. [111] indicate that carbonylation, i.e., ROS-dependent protein modification, could also be triggered by NO. The link between NO, PAs, and ROS seems to be connected with PAs catabolism when H_2_O_2_ is generated via polyamine oxidase activity stimulated by apple embryo treatment with NO [112].

## 5. Maintenance of NO Balance and NO-Dependent Modifications in the Regulation of Seed Physiology

During seed dormancy reversal, changes in NO level were detected for pomegranate (*Punica granatum* L.) seeds and apple embryos [109,113]. NO generation also increased in the embryos of barley seeds, starting from the onset of imbibition [105]. The authors observed that NO levels were higher in seeds of a more dormant cultivar (Sundre), whereas in non-dormant seeds (Harrington) NO production was more rapid. During the culture of seeds of Sundre cultivar, which stayed dormant and did not germinate, even a decrease in NO level in embryos was detected. For Sundre and Harrington seeds, the most significant increase in NO production was observed during the initial stages of germination, when the oxygen conditions in cells become more anaerobic, which may indicate the dominance of the reductive pathway in NO formation (via NO_2_^−^ reduction) [105].

Phytoglobin (Pgb) is responsible for the regulation of NO metabolism [114]. In anoxia, in the Pgb-NO cycle, NO is oxidized to NO_3_^−^ (via oxyPgb), which is further metabolized back into NO_2_^−^ (via NR); this compound is then used for NO formation [114]. During the germination of barley seeds, changes in the expression of gene and protein levels of Pgb were noticed [115]. In embryos of these seeds, Northern blot analysis indicated an increase in *Pgb* transcript levels up to 8 h of imbibition, followed by a decrease in its expression. The enhanced gene expression was the effect of oxygen depletion during seed germination [115]. Research conducted on aleurone layers of barley seeds confirmed the influence of oxygen levels on the expression of *Pgb* genes [116]. After aeration following N_2_ treatment, a decline in *Pgb1*, and *Pgb3* transcripts was observed [116]. Alteration in the expression of *Pgb* was also detected during the germination of barley seeds with varying in dormancy depth [105]. As NO impairs mitochondrial respiration by reversible binding to cytochrome *c* oxidase, Zafari et al. [117] analysed the energy status in barley seeds with up- and downregulated expression of *Pgb*. The upregulation of *Pgb* led to an increased rate of germination and increased ATP/ADP ratios. For seeds with *Pgb* overexpression, a lower rate of NO emission after radicle protrusion (compared to the wild type and seeds with the gene down-expression) and a lower level of the SNO group in proteins were observed in the first hours post imbibition [117]. In seeds under anoxia conditions, the NO-Pgb cycle can be an important alternative to fermentation [116]. In aleurone cells, GA promoted the expression of genes encoding α-amylase, Pgb1 and Pgb3, whereas ABA eliminated the stimulating effects of GA. As GA promotes programmed cell death (PCD), the authors suggested that a decrease in NO levels following GA-induced upregulation of *Pgb* is required to initiate PCD in aleurone layers. In particular, NO inhibits histone deacetylase, the enzyme involved in GA-induced PCD [116] and references herein.

The mechanism of RNS scavenging also includes *S*-nitrosoglutathione reductase (GSNOR) activity. GSNOR is responsible for the denitrosation of *S*-nitrosoglutathione (GSNO, a mobile donor of NO), converting it to oxidized glutathione and ammonia [105,118]. For germinated barley seeds, an increase in GSNOR activity was reported, whereas in non-germinated seeds, it remained at a lower level [105]. In apple embryos, the opposite results were obtained [119]. In the axes of non-dormant, ready-for-germination embryos, GSNOR activity was lower than in the axes of dormant embryos, which can be accounted for by a transient increase in ROS levels [119], exerting a regulatory function on this enzyme [120]. Reduced GSNOR activity was accompanied by an increase in GSNO levels in the tissue [119]. The role of GSNOR in the regulation of seed germination was proven for Burmese grape (*Baccaurea ramiflora* Lour.) seeds under chilling stress. The use of GSNOR inhibitors (dodecanoic acid (DA) and 5-chloro-3-(2-[(4-ethoxyphenyl)ethylamino]-2-oxoethyl)-1H-indole 2-carboxylicacid (C1)) significantly increased the total RNS pool and reduced the germination rate [121].

GSNO transfers NO to a cysteine residue of protein during trans-*S*-nitrosation, and generally, this modification regulates the function of proteins [15,118]. Desiccation of *Antiaris toxicaria* (Lesch.) seeds was accompanied by H_2_O_2_ accumulation, increased protein carbonylation, reduced *S*-nitrosation of antioxidant enzymes and their activities, and decreased seed germination rate [122]. Application of GSNOR inhibitors (DA and C1) before NO exposure promoted *S*-nitrosation of antioxidant enzymes and enhanced NO-induced seed tolerance to desiccation, which was reflected by increased seed germination [122]. On the other hand, NO negatively regulated ABA signalling during the germination of Arabidopsis seeds through *S*-nitrosation of kinases SnRK2.2 and SnRK2.3, components of the ABA transduction pathway [123]. Activation of SnRK2.2 and, most likely, SnRK2.3 by ABA was blocked by the exogenous GSNO. Furthermore, *S*-nitrosation of ABI5 (transcription factor of ABA transduction pathway) marked it for degradation [124]. Recently, Liu et al. [125] presented interesting results using seeds of an Arabidopsis mutant with RNAi silencing *GSNOR1*. The authors proved that NO affected seed oil content and fatty acid composition [125]. For these seeds, reduced GSNOR activity was associated with increased palmitic acid (C16:0), linoleic acid (C18:2), and linolenic acid (C18:3) levels and significantly decreased stearic acid (C18:0), oleic acid (C18:1), and arachidonic acid (C20:1) levels compared to the WT. The effect of NO on the quantity of oil and fatty acids was also confirmed using rapeseed embryos treated with 10 and/or 20 µM SNP [125]. Moreover, proteomics and transcriptome analysis performed in these embryos showed that some *S*-nitrosated proteins and genes involved in oil metabolism were differentially regulated by SNP.

The reaction between NO and O_2_^•−^ results in the formation of ONOO^−^, a nitrating agent. Compounds that can be nitrated include proteins, lipids, and nucleic acids. Protein nitration is connected to the covalent binding of a nitro (–NO_2_) group to one of the two equivalent ortho carbons of tyrosine (Tyr), forming 3-nitro-Tyr [126]. It is considered a stable PTM can thus be used as a reliable marker of nitro-oxidative conditions. Dormancy alleviation and germination of apple embryos were associated with a decrease in protein nitration. The pattern of nitrated proteins suggested that storage proteins were putative targets of nitration, and this PTM acted as an alternative to carbonylation in labelling proteins for degradation [127]. Protein labelling for degradation via the addition of a -NO_2_ group was reported for ABA receptors PYR/PYL/RCAR. Tyr nitration in these proteins resulted in polyubiquitylation and proteasome-dependent degradation [128].

Lipid (fatty acid) nitration is an increasingly studied NO-dependent modification. During the germination of oilseed rape seeds, a decrease in nitro-oleic acid (NO_2_-OA) content was observed [129]. Application of NO_2_-OA significantly increased the NO level and stimulated seed germination. The authors suggested that during seed germination, NO_2_-OA may act as a NO donor. A high content of nitro-linolenic acid, which may contribute to the increased availability of NO, favouring germination, has also been detected in Arabidopsis seeds [130].

Only a few reports have indicated the occurrence of nitrated nucleic acids in plant cells [131,132]. In seeds, increased RNA nitration was observed in the axes of embryos treated with NO [84]. It was assumed that this type of modification that selects mRNA that should not be translated could act as a mechanism related to the transition from dormant to non-dormant state.

**Figure 4:**
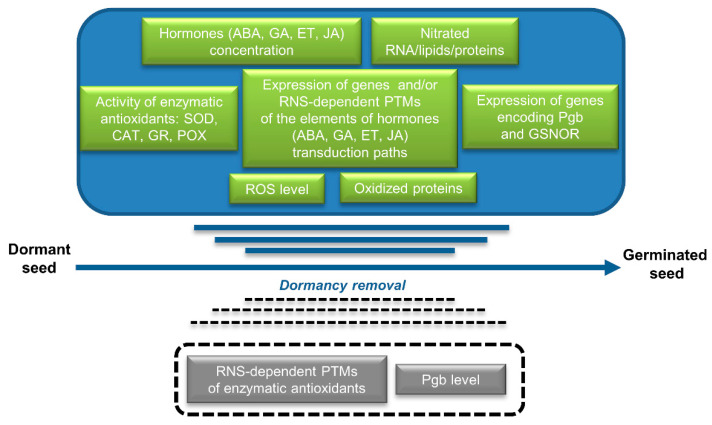
RNS (NO)-induced changes leading to seed dormancy removal and stimulation of seed germination. **The order of changes marked in the figure does not reflect the actual sequence of events**. Predicted but unproven changes are indicated in boxes with dashed lines.

RNS-induced reactions (described in detail in Section 3, Section 4 and Section 5) leading to the transition of seeds from dormant to non-dormant/germination state are shown in Figure 4.

## 6. NO as a Seed Antiaging Molecule

Seed aging is a natural process, related to deleterious effects in cellular compartments manifested by limitations in seed vigour, germination, and seedling development [20,133]. The intensification of harmful processes in seeds occurs during prolonged seed storage under inconvenient conditions, especially at high humidity and high temperature. In laboratory practice, these conditions are used to accelerate aging events and ensure homogeneous material for further analysis.

Recently, attention has been paid to diminishing the negative effects of seed aging, and the role of NO in seed vigour improvement has appeared to be promising [20]. In particular, a decrease in NO level during seed aging was confirmed. Spectrofluorometric analysis of NO production in Siberian elm (*Ulmus pumila* L.) seeds demonstrated an increase in endogenous NO levels during the early stages of aging, which later reduced [134]. Other research has shown that NO release was reduced in the axes of warm-stratified apple seeds compared to the axes of cold-stratified seeds [109]. Cold stratification (5 °C) of apple seeds promotes dormancy alleviation and further germination of the isolated embryos, whereas prolonged warm stratification (25 °C) leads to seed aging [109].

After the application of NO donors, mitigation of aging resulted in vigour improvement, and increased germination was detected for apple embryos and Siberian elm or oat (*Avena sativa* L.) seeds [134,135,136]. In studies published in languages other than English, the positive effect of NO on the germination of aged seeds was confirmed for *Plathymenia reticulata* (Benth) and pumpkin (*Cucurbita pepo* L.) seeds [137,138]. Research conducted by Chakraborty et al. [139] showed a positive effect of KNO_3_ on the quality of stored bottle gourd (*Lagenaria siceraria* (Molina) Standl) seeds. Additionally, priming of aged groundnut (*Arachis hypogaea* L.) seeds with 150 µM SNP before drought stress improved seed germination and seedling vigour through the modulation of oxidative stress [140]. An NO-dependent increase in the quality of the aged *Plathymenia reticulata* seeds was achieved by maintaining the integrity of the membrane and stimulating the activity of SOD [137]. In artificially aged oat seeds, the anti-aging action of NO was also related to the regulation of ROS levels [136]. In the mitochondria of these seeds, a NO-dependent decrease in H_2_O_2_ content was accompanied by an increase in the activities of enzymes of the antioxidant system, promoting the action of the AsA-GSH cycle. On the other hand, it was postulated that the protective role of NO during seed aging was related to enhanced activity of TCA cycle-related enzymes (fumarate hydratase, malate dehydrogenase, and succinate-CoA ligase) and presumed activation of alternative pathways [136]. Pretreatment of Siberian elm seeds with NO followed by seeds being subject to an aging protocol stimulated GSH synthesis in deteriorated seeds, which could reduce ROS production and subsequent cell death [134]. Additionally, the authors proposed that NO may alleviate oxidative stress-induced cell death in aged seeds via activation of ET biosynthesis, and an increase in the expression of genes encoding *S*-adenosyl-l-methionine synthetase and ACC synthase was observed [134].

In cells of aged seeds, ROS accumulation is often correlated with increased content of oxidised lipids, proteins, or nucleic acids [141,142]. In the embryonic axes of artificially aged apple seeds, increased RNA oxidation was observed [135]. Analysis conducted on Arabidopsis seeds has demonstrated the degradation of storage mRNA during aging [143,144]; thus RNA oxidation needs to be analysed as one of the causes of this process. The NO-dependent improvement in the vigour of apple embryos isolated from aged seeds was related to decreased content of oxidized RNA and nitrated RNA in the embryonic axes [135,145]. With the increase in some transcript levels, it was proposed that NO application downregulates the degradation of stored mRNA and/or promotes the synthesis of new mRNA [145], which probably contributes to increased translation and protein abundance. During aging, the decreased protein content could be the result of, e.g., their carbonylation [133] or disturbances in biosynthesis [141]. SNP priming significantly affected total soluble proteins in aged groundnut seeds, and after seeds treatment with 150 µM SNP, the level of these molecules was the highest [140]. Moreover, after the seeds were subjected to drought, the level of total soluble proteins dropped, but SNP treatment diminished the protein loss.

One of the products of the oxidation processes is *meta*-Tyr (*m*-Tyr), the antimetabolite of phenylalanine (Phe) [146,147]. In recent studies, we [145] confirmed the suitablility of *m*-Tyr as a marker of seed aging and demonstrated that an increased *m*-Tyr/Tyr ratio may be related to the loss of seed viability. We also proposed that the mechanism of NO action in seed longevity maintenance depends on the duration of accelerated aging and the biochemical state of the seeds. When seeds were subjected to an aging protocol for a shorter period, in the axes of NO-treated embryos, the *m*-Tyr content was higher compared to seeds that progressed in aging, whereas Phe was at a similar level. Therefore, it was proposed that NO, through the stimulation of ONOO^−^ formation, causes Phe oxidation, resulting in the accumulation of *m*-Tyr. On the other hand, an increase in *m*-Tyr level could be the result of enhanced proteolysis of oxidized proteins. After extending the aging protocol, NO application was correlated with increased content of Phe, whereas the level of *m*-Tyr was similar to that of the untreated embryos. As both amino acids are competitors for tRNA^Phe^, the antiaging action of NO may be associated with the supply of an adequate amount of Phe for the formation of correctly formed proteins [145].

Maintaining the high viability of the seed may be depended on autophagy, a process responsible for recycling the cytoplasmic content. Autophagy can be induced by various pathways, one of which is related to ROS production [148]. Research conducted on aged apple seeds demonstrated the NO-dependent stimulation of transcript levels of genes encoding homologs of NADPH, which is involved in O_2_^•−^ generation [135]. In plants, a target of rapamycin (Tor), an evolutionarily conserved Ser/Thr protein kinase, acts as a negative regulator of autophagy and plays an important role in the control of cell proliferation and growth [149,150,151]. Regardless of the duration of apple seed aging, NO downregulated *Tor* expression in embryonic axes [145]. As Tor also regulates proliferation, we suggested that the prevention of the accumulation of Tor by NO in apple embryos may disrupt this process, minimizing cellular damage [145], in particular because NO simultaneously regulated the expression of genes encoding proteins involved in protein folding, protein structure stabilization, and protein repair. In the embryonic axes of aged seeds, NO treatment did not only impair the expression of genes encoding elements of the Tor complex but probably also affected Tor activity through the regulation of auxin levels. The possibility of a direct impact of NO on Tor activity via PTMs such as nitration or *S*-nitrosation could also be considered [145]. An increase in the content of the SNO group was observed in the early stages of the aging of Siberian elm seeds [134]. Then, the level of SNO decreased rapidly over time but was still higher than that in the unaged seeds. Seed pretreatment with SNP or GSNO before being subject to aging increased the SNO content in the 2-day-aged seeds. Moreover, among 82 *S*-nitrosated proteins in non-aged and aged seeds, 11 were enzymes involved in carbohydrate metabolism. Based on the analysed enzyme activities of carbohydrate metabolism, it seems that NO-mediated protein *S*-nitrosation induces the activation of glycolysis and the inactivation of the pentose phosphate pathway, protecting the seeds from energy deficiency [134].

**Figure 5:**
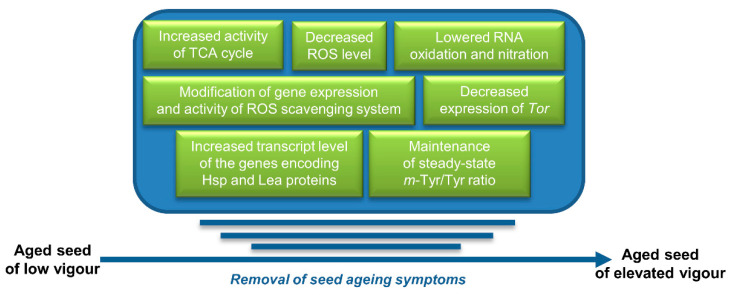
Changes observed after treatment of aged seeds with NO (SNP or other donors) leading to the restoration of seed metabolism, resulting in the removal of typical physiological features of seed aging (low vigour and low germinability). **The course of events indicated in the figure does not reflect their actual sequence**.

Figure 5 shows a summary of RNS-induced modifications leading to the restoration of the metabolism of aged seeds on the literature cited in Section 6.

## 7. Conclusions and Perspectives

In recent years, the processes occurring during RNS (NO)-induced alleviation of seed dormancy have been investigated in depth (Figure 4). Phytohormonal changes concerning the content of hormones and variation in the expression of genes encoding enzymes of biosynthesis and catabolism, as well as genes encoding elements of signal transduction pathways, have been described. Modification of ROS metabolism of both ROS synthesis and scavenging reactions seems to be a key element regulated by NO that can be used as a dormancy-breaking agent. The action of Pgb, in the context of NO-induced dormancy removal and germination, has only been partially elucidated in cereals and in barley seeds with modified expression of Pgb (Figure 4). There is a lack of information on Pgb levels in seeds of other plant species, particularly those characterized by deep embryonic dormancy. ROS- and RNS-dependent modifications of protein, lipids, and nucleic acids may be a primary effect of RNS application, leading to stimulation of seed germination, although no data are available related to RNS-dependent PTMs of enzymes of the cellular antioxidant system.

The use of NO donors in seed-priming and presoaking procedures is still an open question. To date, we have collected only fragmentary knowledge based on very basic, generally biochemical observations (Figure 3). More sophisticated omics studies (proteomic, transcriptomic, and metabolomic) are necessary to fill the gap in our understanding of the events occurring after seed priming with NO or its sources. Taking into account the features of NO donors and compounds released in their decomposition, it seems advisable to seek improved (more specific) sources of NO.

The beneficial effect of NO resulting in the diminishing of aging symptoms in seeds is an interesting observation. The molecular basis of this NO action has been intensely investigated in the past decade, mostly in the context of the regulation of ROS metabolism and ROS-dependent cellular deterioration. Recently, new threats originated from our knowledge on molecular events that explain aging processes in animals have been explored (Figure 5).

## Figures and Tables

**Table 1:** The most commonly used NO donors.

NO Donor	Released Molecules	Conditions of NO Release	References
Sodium nitroprusside (SNP, sodium nitroferricyanide(III) dihydrate)	NO^+^, CN^−^, a mixture of ferrocyanide and ferricyanide products	Photolysis after irradiation with UV-Vis light (except red light). Reaction with reducing agents (e.g., thiols, haemoproteins, and ascorbate) in darkness.	[23,24,25]
RSNO compounds, e.g., *S*-nitroso-N-acetyl-D-penicillamine (SNAP) *S*-nitrosoglutathione (GSNO)	^•^NO, NO^+^, NO^−^, disulphide	Disruption by heat; UV light; and some metal ions, superoxides, and seleno compounds. Metal ions (Cu^+^, Fe^2+^, Hg^2+^, and Ag^+^) serve as important catalysts for the decomposition of the compounds.	[26,27]
3-Morpholinosydnonimine (SIN-1)	^•^NO, O_2_^•−^, ONOO^−^, SIN-1C	Decomposition occurs under alkaline pH and is facilitated by oxygen and Vis light irradiation. In vivo, oxidizing agents stimulate ^•^NO production from SIN-1 at low oxygen concentrations. Under such conditions, SIN-1 is likely to behave more like an ^•^NO donor than an ONOO^−^ donor.	[23,26,28]
Diazeniumdiolates (NONOates), e.g., Diethylenetriamine NONOate (DETA-NONOate, alternative name NOC-18),	^•^NO, NO^+^, NO^−^, NO_2_^−^, nucleophile residue	Decomposition is spontaneous and pH- and temperature-dependent. Dissociation to NO is acid-catalysed, and the rate decreases as the pH increases.	[29,30]
Angeli’ salt (sodium α-oxyhyponitrite, belongs to the NONOate compounds)	NO^−^, ^•^OH, HNO	Spontaneous dissociation in a pH-dependent manner.	[26]
Acidified nitrite	2NO_2_^−^ + 2H^+^⇌ N_2_O_3_ + H_2_O N_2_O_3_⇌ NO_2_ + NO	The reaction requires a high NO_2^−^_ concentration and low pH or the presence of the reductants. e.g., ascorbate. Under less acidic pH, the reduction of NO_2_^−^ to NO is catalysed by metalloproteins (e.g., haemoglobin).	[31,32]
Roussin’s black salt ((KFe_4_S_3_(NO)_7_), the tetra-iron–sulfur-nitrosyl cluster)	NO * and ferric precipitates	Photolysis at λ increases from 313 to 546 nm.	[33]

* The form of NO was not specified.

**Table 2:** NO-dependent changes in the expression of genes related to ABA metabolism and signalling in seeds or siliques.

	Gene	Changes in Gene Expression	Plant Material	General Description of the Material and Results	References
Biosynthesis	*NCED6*	↑/↓	Hedge mustard seeds	Upregulated at 3 h and downregulated at 22 and 26 h of imbibition of non-after-ripened seeds in 20 mM KNO_3_.	[83]
	*NCED9*			Upregulated at 12 h and downregulated at 22 and 26 h of imbibition of non-after-ripened seeds in 20 mM KNO_3_.	
	*NCED5*	↑	Hedge mustard seeds	Among tested *NCED*, *NCED5* expression the highest during imbibition of 7-month after-ripened seeds and the most affected by 20 mM KNO_3_.	[88]
	*NCED6*			Stimulated by 20 mM KNO_3_ only between 22 and 26 h of imbibition of 7-months after-ripened seeds.	
	*NCED9*				
	*NCED3*	↓	Apple seeds	Downregulated in axes of embryos after 3 h of fumigation with vapours of acidified 20 mM NaNO_2_.	[84]
	*NCED9*				
Degradation	*CYP707A2*	↑	Arabidopsis seeds	Increased between 3 and 48 h of imbibition after 200 µM SNP application.	[89]
	*CYP707A1*	↓	Arabidopsis siliques	Downregulated at 15, 19, and 22 d after flowering and was higher in siliques from a mother plant treated with 10 mM NO_3_^−^ compared to the plant treated with 3 mM NO_3_^−^.	[80]
	*CYP707A2*	↑		Increased at 19 and 22 d after flowering and was higher in siliques from a mother plant treated with 10 mM NO_3_^−^ compared to the plant treated with 3 mM NO_3_^−^.	
	*CYP707A2*	↑	Hedge mustard seeds	Upregulated between 3 and 22 h of imbibition of non-after-ripened seeds in 20 mM KNO_3_.	[83]
	*CYP707A2*	↑	Hedge mustard seeds	Increased between 3 and 26 h of imbibition of 7-months after-ripened seeds in 20 mM KNO_3_.	[88]
	*CYP707A1*	=	Apple seeds	Observed in axes of embryos after 3 h of fumigation with vapours of acidified 20 mM NaNO_2_.	[84]
	*CYP707A2*	↓			
Signalling	*RCAR3*	↑	Apple seeds	Observed in axes of embryos after 3 h of fumigation with vapours of acidified 20 mM NaNO_2_.	[84]
	*ABF*	↓			
	*ABI5*	↑/↓	Hedge mustard seeds	Increased at 12 h and decreased at 3 and 26 h of imbibition of non-after-ripened seeds in 20 mM KNO_3_.	[83]
	*ABI5*	↑	Hedge mustard seeds	Stimulated by 20 mM KNO_3_ between 6 and 22 h of imbibition of 7-months after-ripened seeds.	[88]

↑ upregulation, ↓ downregulation, = no changes in gene expression.

**Table 3:** NO-dependent changes in the expression of genes related to GA metabolism and signalling in seeds.

	Gene	Changes in Gene Expression	Plant Material	General Description of the Material and Results	References
Biosynthesis	*GA3ox2*	↓	Hedge mustard seeds	Downregulated at 3, 6, 22, and 26 h of imbibition of non-after-ripened seeds in 20 mM KNO_3_.	[80]
	*GA20ox2*	↑↓		Upregulated only at 3 h and downregulated after 6, 12, and 26 h of imbibition of non-after-ripened seeds in 20 mM KNO_3_.	
	*GA2ox6*	↓		Downregulated after 22 and 26 h of imbibition of non-after-ripened seeds in 20 mM KNO_3_.	
	*GA3ox2*	↑	Hedge mustard seeds	Stimulated by 20 mM KNO_3,_ especially at 22 and 26 h of imbibition of 7-months after-ripened seeds.	[81]
	*GA20ox2*	↑		Upregulated between 6 and 26 h of imbibition of 7-month after-ripened seeds treated with 20 mM KNO_3_.	
	*GA2ox6*	↑			
	*GA3ox1*	↓	Arabidopsis seeds	CN^−^ alleviates the dormancy of Arabidopsis seeds. Seed treatment with 200 µM cPTIO reduced gene expression compared with single CN^−^ application.	[85]
	*GA3ox2*				
Signalling	*RGL2*	↓	Hedge mustard seeds	Downregulated after 22 and 26 h of imbibition of non-after-ripened seeds in 20 mM KNO_3_.	[80]
	*RGL2*	↓	Hedge mustard seeds	In 7-months after-ripened seeds, expression was negatively affected by 20 mM KNO_3_ at 6 and 22 h of imbibition.	[81]

↑ upregulation, ↓ downregulation.

**Table 4:** NO-dependent changes in the expression of genes related to ET metabolism and signalling in seeds.

	Gene	Changes in Gene Expression	Plant Material	General Description of the Material and Results	References
Biosynthesis	*ACS7*	↑	Hedge mustard seeds	After 20 mM KNO_3_ treatment, the *ACS7* transcript was expressed only at the beginning of imbibition (3 h) in seeds that were not after-ripened, and after-ripening eliminated this expression.	[86]
	*ACO2*	↑/↓		After 20 mM KNO_3_ treatment, the transcript level was very high in non-after-ripened seeds at 3 h of imbibition and strongly diminished up to 12 h, increasing afterwards; after-ripening reduced transcript accumulation during the first 6 h of imbibition.	
	*ACS1*	↑	Wild cabbage seeds	Upregulated between 12 and 48 h of imbibition in seeds treated with 5 mM NO.	[72]
	*ACS3*				
	*ACS7*				
	*ACS11*				
	*ACS9*			Increased at 12 and 24 h of imbibition of seeds treated with 5 mM NO.	
	*ACS4*	↓		Decreased between 12 and 48 h of imbibition in seeds treated with 5 mM NO.	
	*ACS5*				
	*ACO1*	↓/↑		Downregulated after 12 h and upregulated after 24 and 36 h of imbibition in seeds treated with 5 mM NO.	
Signalling	*ETR1*	↑	Wild cabbage seeds	Upregulated between 12 and 48 h of imbibition in seeds treated with 5 mM NO.	[72]
	*ETR2*				

↑ upregulation, ↓ downregulation.

**Table 5:** NO-dependent changes in the expression of genes related to JA metabolism and signalling in seeds.

	Gene	Changes in Gene Expression	Plant Material	General Description of the Material and Results	References
Biosynthesis	*AOS1*	↑	Apple seeds	Observed in axes of embryos after 3 h of fumigation with vapours of acidified 20 mM NaNO_2_.	[82]
Derivative formation	*JMT*	↑			
	*JAR1*	↓			
Signalling	*JAZ3*	↓			

↑ upregulation, ↓ downregulation.

## Data Availability

Not applicable.

## Supplementary Materials

The following supporting information can be downloaded at: https://www.mdpi.com/article/10.3390/ijms232314951/s1.

## Abbreviations

^•^OH, hydroxyl radical; ABA, abscisic acid; ACC, aminocyclopropane-1-carboxylic acid; AsA, ascorbic acid; C1, 5-chloro-3-(2-[(4-ethoxyphenyl)ethylamino]-2-oxoethyl)-1H-indole 2-carboxylicacid; CAT, catalase; cPTIO, 2-(4-carboxyphenyl)-4,4,5,5-tetramethylimidazoline-1-oxyl-3 oxide; DA, dodecanoic acid; DETA-NONOate, diethylenetriamine NONOate or N-[bis(2-Aminoethyl)amino]-N-hydroxynitrus amide; DPTA-NONOate, dipropylenetriamine NONOate or (Z)-1-[N-(3-aminopropyl)-N-(3-ammoniopropyl)amino]diazen-1-ium-1,2-diolate; ERFVII, VII ethylene response transcription factor; ET, ethylene; GA, gibberellin; GA2ox, gibberellin 2-oxidase; GA3ox, gibberellin 3-oxidase; GR, glutathione reductase; GSH, glutathione; GSNO, *S*-nitrosoglutathione; GSNOR, *S*-nitrosoglutathione reductase; H_2_O_2_, hydrogen peroxide; Hsp, heat shock protein; JA, jasmonic acid; Lea, late embryogenesis abundant; *m*-Tyr, *meta*-tyrosine; MeJa, methyl jasmonate; NO, nitric oxide; NO^−^, nitroxyl anion; NO^+^, nitrosonium cation; NO^•^, nitric oxide radicle; -NO_2_, nitro group; NO_2_-OA; nitro-oleic acid; NOC, NO-amine complexes, alternative name for DETA-NONOate; NR, nitrate reductase; O_2_^•-^, superoxide anion; ONOO^-^, peroxynitrite; PAs, polyamines; PCD, programmed cell death; Pgb, phytoglobin; POX, peroxidase; PTIO, 2-phenyl-4,4,5,5-tetramethylimidazoline-1-oxyl 3-oxide; PTMs, posttranslational modifications; RNS, reactive nitrogen species; ROS, reactive oxygen species; RSNO, *S*-nitrosothiol; SIN-1, morpholinosydnonimine; SIN-1C, 3-morpholinoiminoacetonitrile; SNAP, *S*-nitroso-N-acetyl-D-penicillamine; SNO, *S*-nitrosated groups; SNP, sodium nitroprusside; SOD, superoxide dismutase; TCA, tricarboxylic acid; Tor, target of rapamycin; Tyr, tyrosine.
