# Supplementary material for: Nitric Oxide in Seed Biology
Source: Int J Mol Sci. 2022 Nov 29;23(23):14951. doi: 10.3390/ijms232314951 (PMC9736209; doi:10.3390/ijms232314951)
Supplement: Supplementary file 1 [file ijms-23-14951-s001.zip › ijms-2021733-supplementary.pdf]

Supplementary materials

Database search details

On August 11, 2022, a literature search was performed with the Web of Science (WoS) online database (https://www.webofscience.com/) to identify papers with the following search strategy:

1. Search for papers on nitric oxide in plants: (“reactive+nitrogen+species” OR “RNS” OR “nitrosative+stress” OR “nitric+oxide”) AND ("plant") OR ("plants"). This query searched for papers that contain the word reactive nitrogen species or RNS or nitrosative stress or nitric oxide and plant or its derivatives in their title, abstract, or keywords.

2. Search for papers on nitric oxide and seeds: (“reactive+nitrogen+species” OR “RNS” OR ”nitrosative+stress” OR “nitric+oxide”) AND ("seed") OR ("seeds"). This query searched for papers that contain the word reactive nitrogen species or RNS or nitrosative stress or nitric oxide and seed or seeds in their title, abstract, or keywords.

No restrictions were imposed on publication type (e.g., original article, review, and editorial), or publication language. Publication years were restricted from 1900 to 2021. Results were filtered by publication year or Web of Science Categories.

Table S1. Count of papers on NO in plant science assigned to the Web of Science Categories

**Web of Science Categories**	**Record Count**	**Record ratio (% of 16 263)**
Plant Sciences	6053	37.2
Biochemistry Molecular Biology	2954	18.2
Pharmacology Pharmacy	2865	17.6
Chemistry Medicinal	1903	11.7
Integrative Complementary Medicine	1071	6.6
Food Science Technology	1059	6.5
Cell Biology	818	5.0
Environmental Sciences	808	4.9
Chemistry Multidisciplinary	751	4.6
Biotechnology Applied Microbiology	622	3.8

The table shows 10 top-scored results

Table S2. Count of papers on NO and seeds assigned to the Web of Science Categories

**Web of Science Categories**	**Record Count**	**Record ratio (% of 3349)**
Plant Sciences	980	29.3
Biochemistry Molecular Biology	573	17.1
Pharmacology Pharmacy	428	12.8
Food Science Technology	322	9.6
Chemistry Medicinal	287	8.6
Cell Biology	183	5.5
Chemistry Multidisciplinary	158	4.7
Nutrition Dietetics	152	4.5
Environmental Sciences	141	4.2
Integrative Complementary Medicine	128	3.8

The table shows 10 top-scored results
